# Statistical analysis plans (SAPS) for academic clinical trials at the edinburgh clinical trials unit: what should they contain?

**DOI:** 10.1186/1745-6215-14-S1-O102

**Published:** 2013-11-29

**Authors:** Aryelly Rodriguez, Steff Lewis, Gordon Murray, Ashma Krishan, Isabella Butcher, Christopher Weir

**Affiliations:** 1 Centre for Population Health Sciences-University of Edinburgh, Edinburgh, UK; 2 ECTU-University of Edinburgh, Edinburgh, UK; 3 HSRU, Edinburgh, UK

## 

Once a study protocol is final, the creation of the Statistical Analysis Plan (SAP) can commence. Accordingly to ICH E9 a SAP "contains a more technical and detailed elaboration of the principal features of the analysis described in the protocol" but the level of detail is not specified. This topic is currently being discussed between UK-CRC registered Trials Units, and there is as yet no agreement on the amount of detail that should be covered in the SAP. Within the Edinburgh Clinical Trials Unit (ECTU), we created a consensus document outlining what we thought the essential aspects of a SAP should be.

Briefly, we set out the essential aspects of a SAP as follows. It should be written by someone who has not seen unblinded data from the trial, and should describe analyses unambiguously and in sufficient detail for another statistician to be able to repeat them. It should include a brief description of the trial design, and the statistical methods section from the protocol. Analysis populations, e.g. ITT, per protocol, should be defined in an unambiguous fashion. The SAP should state overall level of significance, and any other relevant information that will be used in the majority of analyses (e.g. treatment of missing data, adjustment for covariates, adjustment for multiplicity, adjustment of p-value due to interim analyses). A list of analyses should describe each precise outcome, the analysis method to be used, any sensitivity analyses, any deviations from the methods listed in ‘Overall Statistical Principles', and any subgroup analyses.

